# An exceptionally late and severe radiation recall dermatitis with chest-wall ulceration 32 years after breast radiotherapy: a case report and literature review

**DOI:** 10.1007/s13691-026-00885-z

**Published:** 2026-07-24

**Authors:** Reina Kamohara, Mai Okazaki, Aya Ueda-Sawa, Riko Sato, Tomohei Matsuo, Sachie Hashimoto, Akiko Iguchi-Manaka, Hiroko Bando

**Affiliations:** 1 Department of Breast-Thyroid-Endocrine Surgery, University of Tsukuba Hospital, 1-1-1 Tennodai, 305-8575 Tsukuba, Ibaraki Japan; 2 Department of Breast Surgery, Ibaraki Prefectural Central Hospital, Ibaraki Cancer Center, Kasama, Ibaraki Japan; 3 Department of Breast and Endocrine Surgery, Institute of Medicine, University of Tsukuba, Tsukuba, Ibaraki Japan

**Keywords:** Radiation recall phenomenon, Breast cancer, Late-onset, Docetaxel, Carboplatin

## Abstract

Radiation recall phenomenon (RRP) is an inflammatory reaction occurring in previously irradiated tissues after exposure to certain drugs, and very late-onset cases are exceptionally rare. We report a 58-year-old woman with Human Epidermal Growth Factor Receptor 2 (HER2)-positive, estrogen receptor (ER) / progesterone receptor (PgR)-positive invasive ductal carcinoma of the left breast who had undergone contralateral mastectomy and radiotherapy 32 years earlier. During neoadjuvant docetaxel, carboplatin, trastuzumab, and pertuzumab, she developed rapidly progressive ulceration and necrosis confined to the prior radiation field on the right chest wall. Docetaxel and carboplatin were discontinued while trastuzumab and pertuzumab were continued. Intensive dermatologic management, including repeated surgical debridement and topical therapy, was required, and substantial epithelialization was achieved only after an approximately two-year course, highlighting a protracted recovery. The clinical course was considered most consistent with severe late-onset radiation recall dermatitis (RRD) superimposed on chronically vulnerable irradiated tissue. Clinicians should consider RRD during systemic therapy in patients with a history of radiotherapy, even decades after treatment, and should distinguish it from chronic radiation injury, infection, and recurrent cancer.

## Introduction

Radiation recall phenomenon (RRP) is a drug-triggered inflammatory reaction that reappears within previously irradiated tissue, most commonly as dermatitis sharply limited to the radiation field. Most cases occur within weeks to months after radiotherapy, whereas reactions arising years to decades later are rare [1, 2]. Because long-term cancer survivors increasingly receive additional systemic therapy, recognition of delayed radiation recall has become clinically relevant. However, in very late presentations, differentiating radiation recall dermatitis (RRD) from chronic radiation injury, infection, or recurrent malignancy can be difficult. We report an exceptionally late and severe chest-wall RRD with ulceration occurring 32 years after breast radiotherapy during neoadjuvant treatment for a new contralateral breast cancer, together with a review of reported cases occurring more than 10 years after radiotherapy.

## Case report

**Fig. 1:**
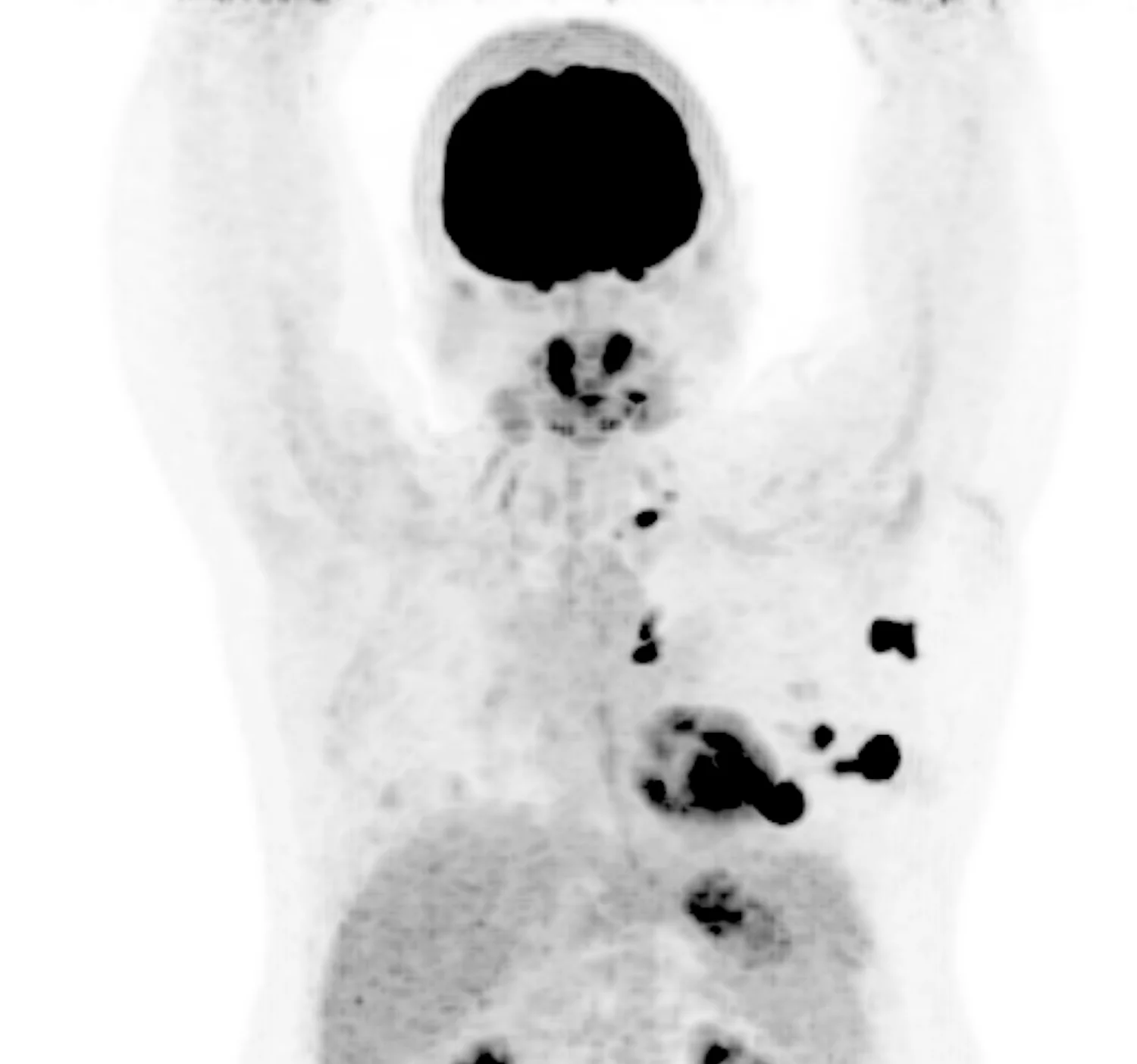
18 F-FDG PET/CT showing intense uptake in the primary tumor of the left breast and in left axillary level I–III, internal mammary, and supraclavicular lymph nodes

A 58-year-old woman presented with left breast cancer. Core needle biopsy revealed histological grade II invasive ductal carcinoma (IDC), estrogen receptor (ER)-positive, progesterone receptor (PgR)-positive, and Human Epidermal Growth Factor Receptor 2 (HER2)-positive (IHC 3+), with a Ki-67 index of 30–40%. Positron emission tomography /computed tomography (PET/CT) demonstrated uptake of 18 F-fluorodeoxyglucose (FDG) in axillary, internal mammary, and supraclavicular lymph nodes (Fig. 1). The clinical stage was cT2N3cM0, stage IIIC. The patient had a body mass index of 32 and a medical history marked by contralateral breast cancer and pregnancy termination. Otherwise, her medical history was unremarkable.

**Fig. 2:**
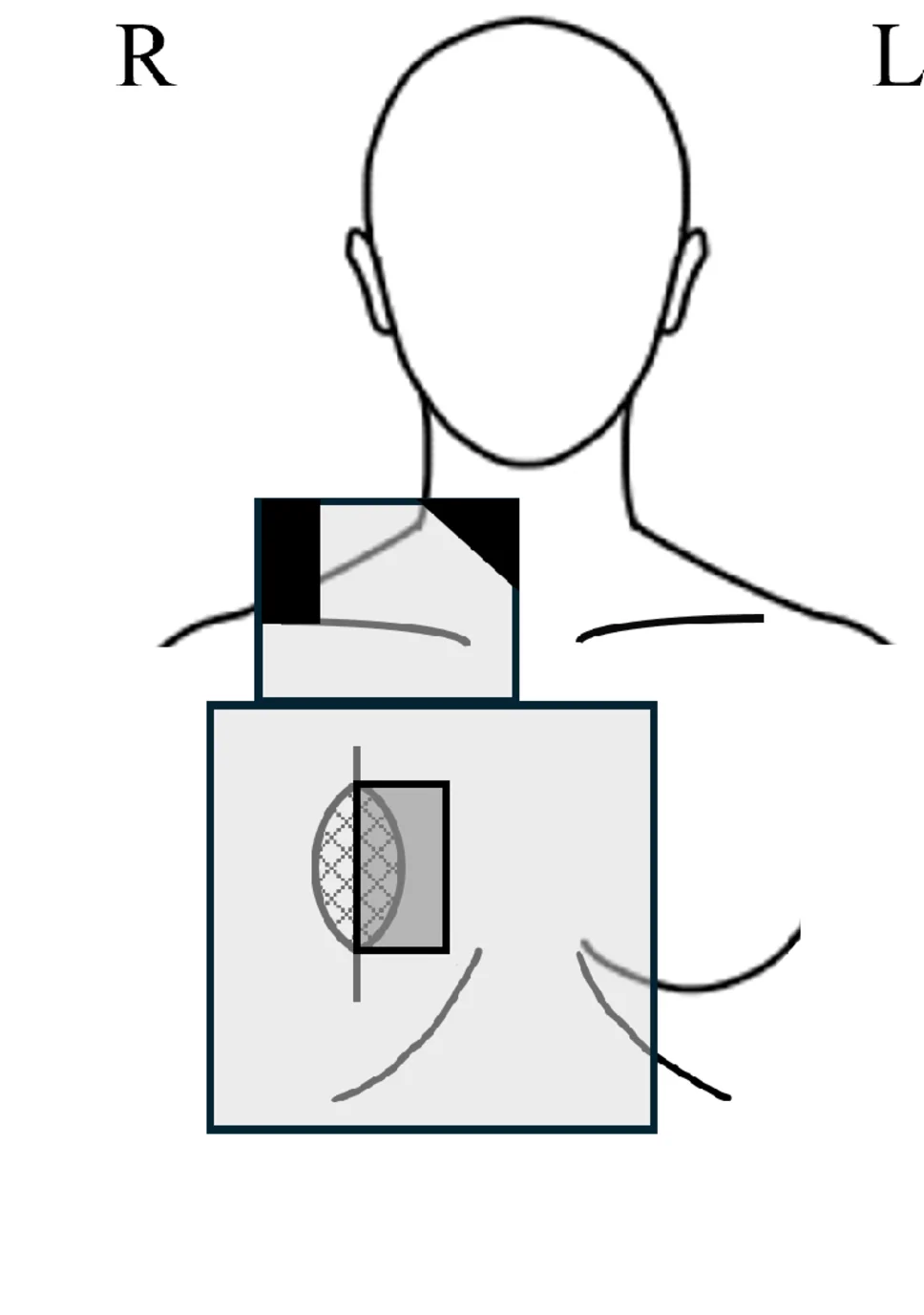
The schematic illustration of right-sided chest wall irradiation field. This has been reconstructed from handwritten medical records, since the original radiotherapy planning images were not available. The light-grey area indicates cobalt irradiation to the right chest wall – parasternal region and right supraclavicular fossa (50 Gy in 25 fractions). The dark-grey area indicates an electron boost to the area around the positive margin near the surgical/graft site (15 Gy in 6 fractions). The hatched oval indicates the full-thickness skin graft, and the vertical lines represent the wound edges. Black-filled areas are presumed to be shielded/trimmed regions

Thirty-two years earlier, she had stage IIIC right breast cancer and underwent right extended radical mastectomy with full-thickness skin grafting. Pathology showed IDC, ER-positive, HER2-negative, with a positive medial margin (T2N3c, stage IIIC). She received postoperative radiotherapy starting four weeks after surgery, delivered with cobalt units to the right chest wall–parasternal region and right supraclavicular fossa (50 Gy in 25 fractions), followed by an electron boost of 15 Gy in 6 fractions to the area around the positive margin near the surgical/graft site (Fig. 2). She then received oral doxifluridine and tamoxifen, which were both discontinued due to hepatotoxicity Subsequent genetic testing identified a pathogenic *BRCA2* variant.

**Fig. 3:**
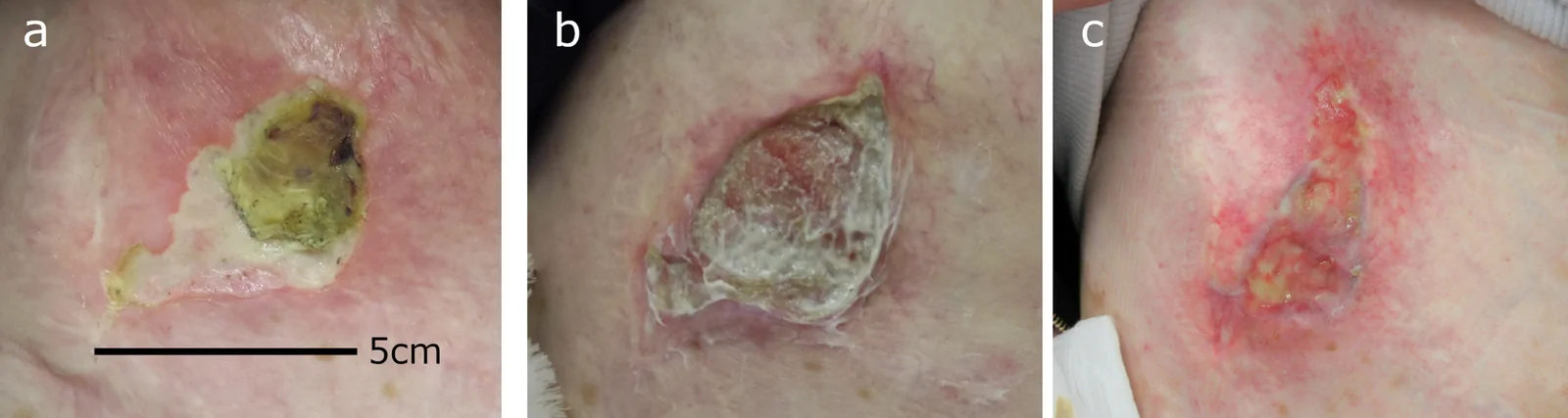
Sequential clinical photographs of the right chest-wall lesion: (**a**) before the fourth cycle of TCbHP, (**b**) after discontinuation of docetaxel/carboplatin with continuation of trastuzumab/pertuzumab for two cycles, and (**c**) gradual epithelialization approximately two years after onset

**Fig. 4:**
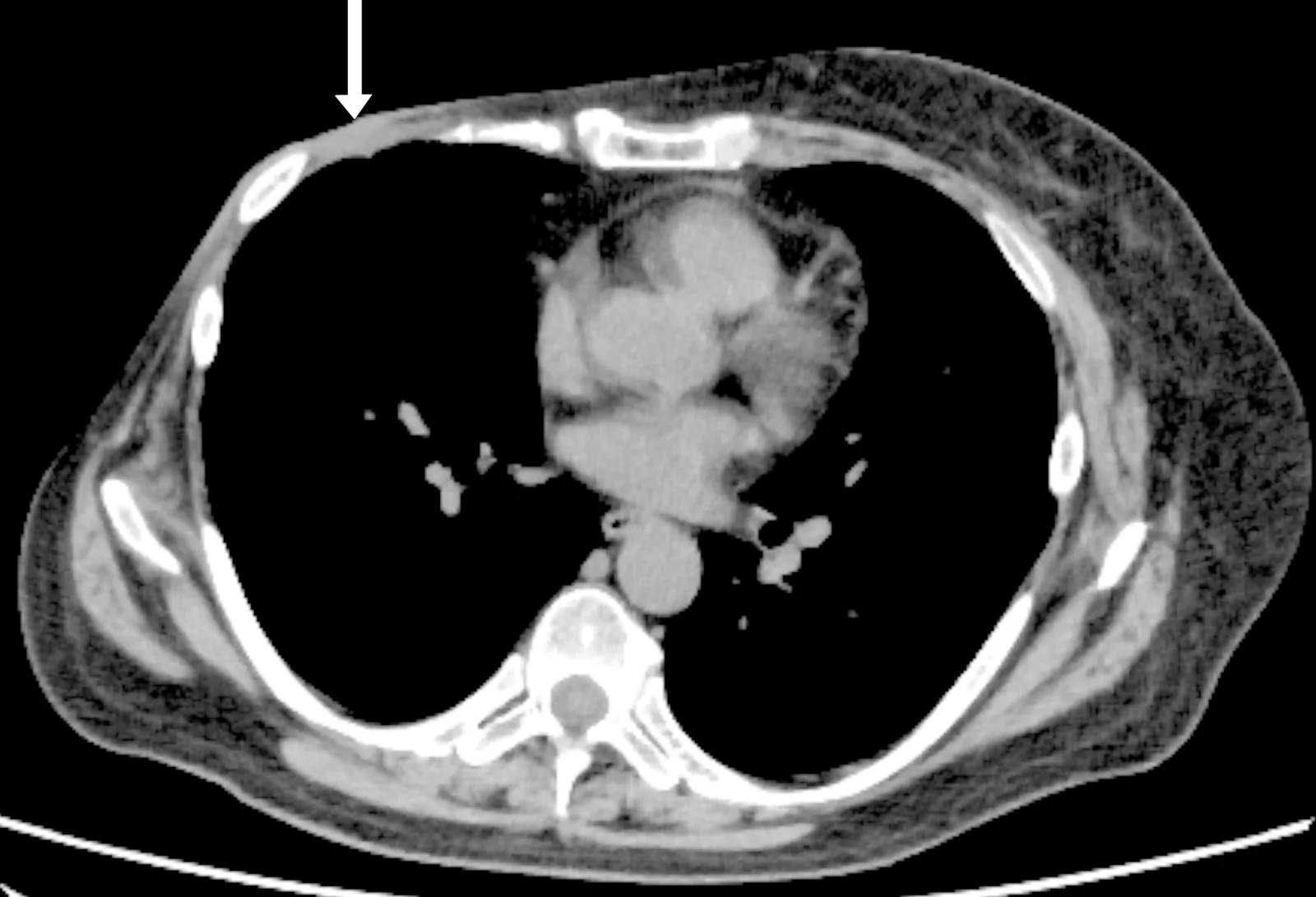
Axial chest CT showing soft-tissue change in the skin and subcutaneous tissue around the right sixth rib corresponding to the clinically observed ulcer, without an obvious mass suggestive of chest-wall recurrence

For the current left breast cancer, neoadjuvant chemotherapy with docetaxel, carboplatin, trastuzumab, and pertuzumab (TCbHP) was initiated. After three cycles, the patient developed grade 2 diarrhea, necessitating a dose reduction of carboplatin. Seventeen days after the third cycle, at the pre-treatment evaluation before the fourth cycle, a rapidly progressive ulcer with necrosis localized to the previously irradiated right chest wall was observed (Figs. 3a and 4). The lesion was considered most consistent with late-onset RRD rather than simple progression of chronic radiation injury. This interpretation was based on the abrupt onset during active systemic therapy, the sharp confinement to the prior radiation field, the absence of other apparent triggers such as recurrent malignancy, overt infection, or trauma, and subsequent stabilization after withdrawal of docetaxel and carboplatin. Chest computed tomography showed soft-tissue changes corresponding to the ulcer (Fig. 4). Despite the absence of histopathologic confirmation, there were no findings that exhibited a high degree of suggestiveness for chest-wall recurrence. The overall temporal and spatial pattern supported a recall reaction.

Dermatologic management included repeated debridement and topical application of silver sulfadiazine and corticosteroid ointments. Docetaxel and carboplatin were discontinued, while trastuzumab and pertuzumab were continued for two additional cycles. Because taxanes are among the most commonly reported triggers of RRD, docetaxel was considered the most likely causative agent. Carboplatin was regarded as a possible contributing factor, whereas continued trastuzumab and pertuzumab without further worsening made these agents less likely to be the principal trigger, although an additive effect on impaired wound healing cannot be excluded. During this period, no exacerbation of the ulcer was observed (Fig. 3b). The patient subsequently underwent left mastectomy with axillary dissection, and the pathological stage was ypT3N1a, stage IIIA. Postoperatively, she underwent external-beam radiotherapy to the left chest wall and left supraclavicular region (50 Gy in 25 fractions) with an additional boost to the left supraclavicular region (10 Gy in 5 fractions). The left chest-wall fields were planned to spare the midline as much as feasible because the parasternal region had been irradiated during treatment for the prior right breast cancer. Subsequently, she underwent CyberKnife stereotactic radiotherapy directed to the parasternal/internal mammary nodal region (50 Gy in 25 fractions), with an additional boost of 10 Gy in 5 fractions to the parasternal lymph node that had shown FDG uptake before neoadjuvant chemotherapy. She continued letrozole in combination with trastuzumab and pertuzumab. No recurrence of the chest-wall ulcer was observed during postoperative therapy, and gradual epithelialization was confirmed at the two-year follow-up from onset (Fig. 3c).

## Discussion

RRP was first described by D’Angio et al. in 1959 [3]. It is characterized by an inflammatory reaction in previously irradiated tissues triggered by systemic agents. The pathogenesis remains uncertain, with proposed mechanisms including vascular injury, epithelial stem-cell depletion, altered local cytokine responses, drug hypersensitivity, and non-immune inflammatory activation. Cytotoxic chemotherapy is the most frequent precipitant, but RRP has also been reported with molecular-targeted agents, immune checkpoint inhibitors, antibiotics, and even COVID-19 vaccines. Reported incidence varies widely, ranging from 1.8% to 11.5%, partly because diagnostic criteria are not standardized and milder cases may go unrecognized [1, 2, 4].

Typical onset occurs within weeks to months after radiotherapy. The skin is the most commonly affected site, although RRP may also involve lung, muscle, gastrointestinal tract, and other tissues. Clinical severity ranges from mild erythema and desquamation to ulceration and necrosis.

Management generally includes withdrawal of the suspected triggering agent, topical or systemic corticosteroids, wound care, and treatment of complications; selected patients may later undergo cautious rechallenge, defined as re-administration of the suspected causative drug, under close observation [1, 2, 4–7].

Our case is notable for both the extraordinary latency of 32 years and the severity of the presentation. Previously reported cases of radiation recall occurring more than 10 years after radiotherapy identified through a PubMed search are summarized in Table 1 [8–13]. These reports suggest that very late-onset reactions are exceedingly rare and, in most cases, relatively limited in severity. In contrast, our patient developed rapidly progressive ulceration requiring prolonged local management over approximately two years. The historical radiotherapy regimen consisted of 50 Gy in 25 fractions to the chest wall and regional fields, followed by an electron boost of 15 Gy in 6 fractions to the surgical/graft bed. However, robust evidence is lacking regarding how radiation dose relates to the latency, protracted course, or severity of RRP/RRD. In lieu of a solitary dose, the interaction between radiation dose and the interval to subsequent systemic drug exposure has been posited as a factor in influencing risk and clinical behaviour [4]. Furthermore, cases that manifest very late in the course of the disease have demonstrated decades-long latency despite heterogeneous dose regimens, indicating that the radiation dose itself is unlikely to fully explain extreme delays. Furthermore, the present case also highlights a central diagnostic issue: very late ulceration in an irradiated field can easily be attributed to chronic radiation injury alone. In our patient, however, the abrupt onset during TCbHP therapy, the sharp confinement to the historical radiation field, and the plateau after discontinuation of docetaxel/carboplatin favored superimposed RRD on a background of chronically fragile irradiated tissue rather than isolated late radiation ulceration.

**Table 1:** Late-onset cases of RRD that occurred more than 10 years after the last radiotherapy treatment

Report	Primary disease	Radiation dose to lesion (Gy)	Suspected trigger	Interval from RT to recall	Interval from drug exposure to recall	Site	Clinical manifestation	Management	Time to improvement
Koshiro N et al. [8]	Uterocervical carcinoma	50 Gy	Gemcitabine	37 years	13 days	Rectal wall	Rectal bleeding	Observation	2 days
Waled B et al. [9]	Arm soft-tissue tumor of Unknown etiology	Unspecified	Gemcitabine	66 years	8 days	Cutaneous	Erythematous, coalescent patchy rash	Topical triamcinolone, oral antihistamines	3 weeks
Fabrice B et al. [10]	Breast cancer	Unspecified	Pemetrexed	25 years	3 days	Cutaneous	Pain, induration	Systemic steroid	2 weeks
J Burdon et al. [11]	Localized reticulum cell sarcoma of the palate	32.35 Gy	Doxorubicin, Cyclophosphamide, Vincristine sulfate, Methotrexate	15 years	2 weeks	Oral mucosa	Stomatitis	Topical amphotericin B, nystatin	7 weeks
Richard P et al. [12]	Breast cancer	60.4 Gy	Cyclophosphamide, Methotrexate, Fluorouracil	10 years	7 days	Cutaneous	Erythema	Topical steroids	Unknown
Muhammad F et al. [13]	Breast cancer	45 Gy	Spontaneously	40 years	-	Cutaneous	Rash, burning sensation, mild soreness	Topical steroid	6 weeks
Present case	Breast cancer	65 Gy	Docetaxel, Carboplatin,	32 years	17 days	Cutaneous	Necrosis, ulceration	Surgical debridement, silver sulfadiazine, topical steroid	2 years

Docetaxel was considered the most plausible trigger because taxanes are among the best documented causes of radiation recall dermatitis. Nevertheless, causality cannot be established with certainty because carboplatin was co-administered and anti-HER2 therapy was continued. Trastuzumab has been reported to exert indirect anti-angiogenic effects through Vascular Endothelial Growth Factor (VEGF)-related pathways [14, 15], and we therefore cannot exclude the possibility that concomitant HER2-targeted therapy contributed to delayed wound healing or amplified tissue injury rather than initiating the recall event itself. Furthermore, ulceration manifested within the grafted skin that had previously undergone irradiation, indicating that prolonged structural vulnerability and limited local perfusion may have intensified the reaction.

Although surgical debridement and reconstruction were considered, the patient preferred conservative management. Given the extremely limited subcutaneous tissue overlying bone at the grafted site—and the likely consequent restriction in local perfusion—surgical intervention to obtain well-vascularized coverage may be considered in similar settings, particularly when ulceration progresses or when secondary infection or osteitis is suspected. However, it remains unclear whether earlier surgery reliably shortens the treatment course, as severe cases have progressed despite corticosteroid therapy and plastic surgical management [6]. Decisions regarding surgery should therefore be individualized according to lesion depth, infection risk, anatomic feasibility, and patient preference.

The possible influence of *BRCA2* pathogenic variants on susceptibility to RRP is uncertain. *BRCA2* is involved in homologous recombination-mediated DNA repair, but available clinical data do not show clearly increased acute or late radiation toxicity in *BRCA1/2* carriers compared with non-carriers [16]. In the present case, *BRCA2* status is therefore best regarded as a background characteristic rather than evidence of a causal association with RRD.

This report describes a very rare case of late-onset RRD resulting in skin ulceration and prolonged recovery. The main clinical message is that radiation recall should remain in the differential diagnosis for sharply localized inflammatory or ulcerative lesions arising within a prior radiation field, even decades after radiotherapy. Careful assessment is required to distinguish recall from chronic radiation injury, infection, and recurrent malignancy, because management hinges on recognition of the potential drug trigger and appropriate wound care.
